# Systematic screening identifies a 2‐gene signature as a high‐potential prognostic marker of undifferentiated pleomorphic sarcoma/myxofibrosarcoma

**DOI:** 10.1111/jcmm.14814

**Published:** 2019-11-19

**Authors:** Qinsheng Hu, Shijie Zhou, Xuefeng Hu, Hua Zhang, Shishu Huang, Yunbing Wang

**Affiliations:** ^1^ Department of Orthopaedic Surgery National Clinical Research Center for Geriatrics West China Hospital Sichuan University Chengdu China; ^2^ Cancer Center West China Hospital Sichuan University Chengdu China; ^3^ National Engineering Research Center for Biomaterials Sichuan University Chengdu China; ^4^ Department of Orthopedic Surgery and Orthopedic Research Institute West China Hospital Sichuan University Chengdu China

**Keywords:** *ITGA10*, myxofibrosarcoma, *PPP2R2B*, prognosis, undifferentiated pleomorphic sarcoma

## Abstract

The Cancer Genome Atlas (TCGA) Research Network confirmed that undifferentiated pleomorphic sarcoma (UPS) and myxofibrosarcoma (MFS) share a high level of genomic similarities and fall into a single spectrum of tumour. However, no molecular prognostic biomarkers have been identified in UPS/MFS. In this study, by extracting data from TCGA‐Sarcoma (SARC), we explored relapse‐related genes, their prognostic value and possible mechanisms of the dysregulations. After systematic screening, *ITGA10* and *PPP2R2B* were included to construct a 2‐gene signature. The 2‐gene signature had an AUC value of 0.83 and had an independent prognostic value in relapse‐free survival (RFS) (HR: 2.966, 95%CI: 1.995‐4.410 *P* < .001), and disease‐specific survival (DSS) (HR: 2.283, 95%CI: 1.358‐3.835, *P* = .002), as a continuous variable. Gene‐level copy number alterations (CNAs) were irrelevant to their dysregulation. Two CpG sites (cg15585341 and cg04126335) around the promoter of *ITGA10* showed strong negative correlations with *ITGA10* expression (Pearson's *r* < −0.6). Transcript preference was observed in *PPP2R2B* expression. The methylation of some CpG sites in two gene body regions showed at least moderate positive correlations (Pearson's *r* > .4) with *PPP2R2B* expression. Besides, the 2‐gene signature showed a moderate negative correlation with CD4 + T cell infiltration. High‐level CD4 + T cell infiltration and neutrophil infiltration were associated with significantly better RFS. Based on these findings, we infer that the 2‐gene signature might be a potential prognostic marker in patients with UPS/MFS. Considering the potential benefits of immunotherapy for UPS/MFS patients, it is imperative to explore the predictive value of this signature in immunotherapeutic responses in the future.

## 1 INTRODUCTION

Soft tissue sarcomas (STS) are a heterogeneous group of rare tumours with the mesenchymal origin.^1^ Undifferentiated pleomorphic sarcoma (UPS) and myxofibrosarcoma (MFS) are two highly similar and the most common histological subtypes of STS.^2^ Both affect the elderly population and their exact origin is still controversial and have not been proved.^2^ Historically, MFS was originally considered as a subset of UPS, but was reclassified as a distinct entity in 2002 WHO classification due to its clinicopathological characteristics. One recent study by The Cancer Genome Atlas (TCGA) explored their genetic/epigenetic profiles and indicated that they are not distinct tumours, but rather belong to a single spectrum of tumour, due to high similarities in somatic copy number alterations (SCNAs), methylation, miRNA expression and protein expression.^3^ Therefore, common systematic therapeutic strategies might be appropriate.

Currently, surgical resection with radiotherapy is still the most effective strategy for patients with non‐metastatic tumours.^2^, ^4^ However, most UPS is usually associated with deep‐seated lesions that grow aggressively, while MFS is usually characterized by infiltrative growth.^4^, ^5^ Although the five‐year overall‐survival (OS) rate was around 70% in UPS/MFS,^6^, ^7^ they have a high risk of local recurrence and subsequent poor prognosis.^4^, ^5^ Currently, prognosis prediction largely relies on the clinicopathological features, such as age, tumour size, margin status and the presence of infiltrative growth.^8^, ^9^, ^10^ However, no molecular prognostic biomarkers have been identified in UPS/MFS and thus it is quite necessary to analyse their molecular features and to explore potential reliable prognostic biomarkers.

In this study, by extracting the genomic and survival data of UPS/MFS from TCGA‐Sarcoma (SARC), we explored relapse‐related genes, their prognostic value and possible mechanisms of the dysregulations.

## 2 MATERIALS AND METHODS

### 2.1 Retrospective analysis using data from TCGA

Genomic data in TCGA‐SARC, including RNA‐seq (IlluminaHiSeq) data of gene expression, gene‐level copy number alterations (CNAs), somatic mutations and gene‐level DNA methylation data, were obtained using UCSC Xena browser (https://xenabrowser.net/). 11 The part of data from patients with UPS/MFS (N = 61) was further extracted. The updated clinicopathological and survival data were obtained from one previous publication of TCGA Research Network.^3^

RNA‐seq data were normalized and represented by log_2_(norm count + 1), in which the norm_count refers to RSEM value. Gene‐level CNAs were calculated by the method of Genomic Identification of Significant Targets in Cancer 2.0 (GISTIC2),^12^ in which the alterations were defined as homozygous deletion (−2), heterozygous loss (−1), copy neutral (0), low‐level copy gain (+1) and high‐level amplification (+2). Somatic mutations detected include single‐nucleotide polymorphisms (SNPs) and small insertions and deletions (INDELs). Gene‐level DNA methylation was detected by Infinium Human Methylation 450 Bead Chip and the methylation status of individual CpG site was calculated and showed by the β value.

The clinicopathological and survival data collected include age at initial diagnosis, gender, pathological tumour size (mm), adjuvant radiation treatment, adjuvant pharmaceutical drug, Fédération Nationale des Centres de Lutte Contre le Cancer (FNCLCC) grade, relapse status, relapse‐free survival (RFS) time (in days), disease‐specific survival (DSS) status and time (in days).

### 2.2 Receiver operating characteristic (ROC) curve analysis

ROC curve was generated to evaluate the prognostic value of gene expression in terms of relapse and DSS. Area under the curve (AUC) was calculated to assess the potential prognostic value via the following criteria: 0.50‐0.60/0.4‐0.5 = fail, 0.60‐0.70/0.3‐0.4 = poor, 0.70‐0.80/0.2‐0.3 = fair, 0.80‐0.90/0.1‐0.2 = good and 0.90‐1/0‐0.1 = excellent.^13^

### 2.3 Comparison of gene transcripts in sarcoma and normal human tissues

The alternative transcripts of the target genes in sarcoma tissues and normal issues with known positive gene expression were examined using the transcript analysis in UCSC Xena browser (https://xenabrowser.net/)11. Transcript data in normal tissues were obtained from The Genotype‐Tissue Expression (GTEx) project, which contains tissue‐specific transcriptional data based on a large number of samples.^14^, ^15^ Log_2_Transcript per Million (TPM) was calculated and compared.

### 2.4 Construction of potential prognostic gene signatures

To contracture prognostic gene signature, only the genes with independent prognostic value were included for model construction. The weight coefficients of the genes were derived from the regression coefficients in multivariate survival analysis, by setting RFS as the dependent variable. Therefore, the risk score is calculated by the following formula:

$$\text{risk score}=\sum_{i=1}^{n}\text{Bgene}\left(i\right)*\text{Expression gene}\left(i\right),$$

where B refers to the regression coefficient in multivariate survival analysis.

### 2.5 Estimation of immune cell infiltration

The tumour immune cell infiltration in UPS/MFS was estimated using data provided by the Tumor Immune Estimation Resource (TIMER; cistrome.shinyapps.io/timer), which is a web‐based platform that provides the abundance of six tumour‐infiltrating immune subsets (B cells, CD4 + T cells, CD8 + T cells, neutrophils, macrophages and dendritic cells) based on data from TCGA.^16^

### 2.6 Analysis of prognosis‐related genes across different cancer types

The potential prognostic significance of the signature genes in other cancers was explored with the use of GEPIA2 (http://gepia2.cancer-pku.cn/#index), which is a web server for large‐scale expression profiling and interactive analysis.^17^ Prognostic value in terms of RFS and OS was assessed by setting median gene expression as the cut‐off and the log‐rank test. The cancer type with *P*‐value < .05 was highlighted.

### 2.7 Statistical analysis

Data integration and statistical analysis were performed using GraphPad Prism 8.10 (GraphPad Inc) and SPSS Statistics 25.0 (SPSS Inc) Violin plot charts were generated for group comparison. For multiple group comparison, one‐way ANOVA with post hoc Tukey's multiple comparisons test was conducted, while the comparison between two groups was performed using Welch's unequal variances t test.

Kaplan‐Meier (K‐M) curves of RFS/DSS were generated for prognostic comparisons, with log‐rank test to detect the difference. Univariate and multivariate Cox regression (method, forward: LR) models were used to evaluate the independent prognostic value of the 2‐gene signature as a continuous variable, regarding RFS and DSS, respectively. Pearson's correlation coefficients were calculated to estimate correlation. *P* < .05 was considered statistically significant.

## 3 RESULTS

### 3.1 Systematic screening to identify relapse‐related genes in UPS/MFS

**Figure 1:**
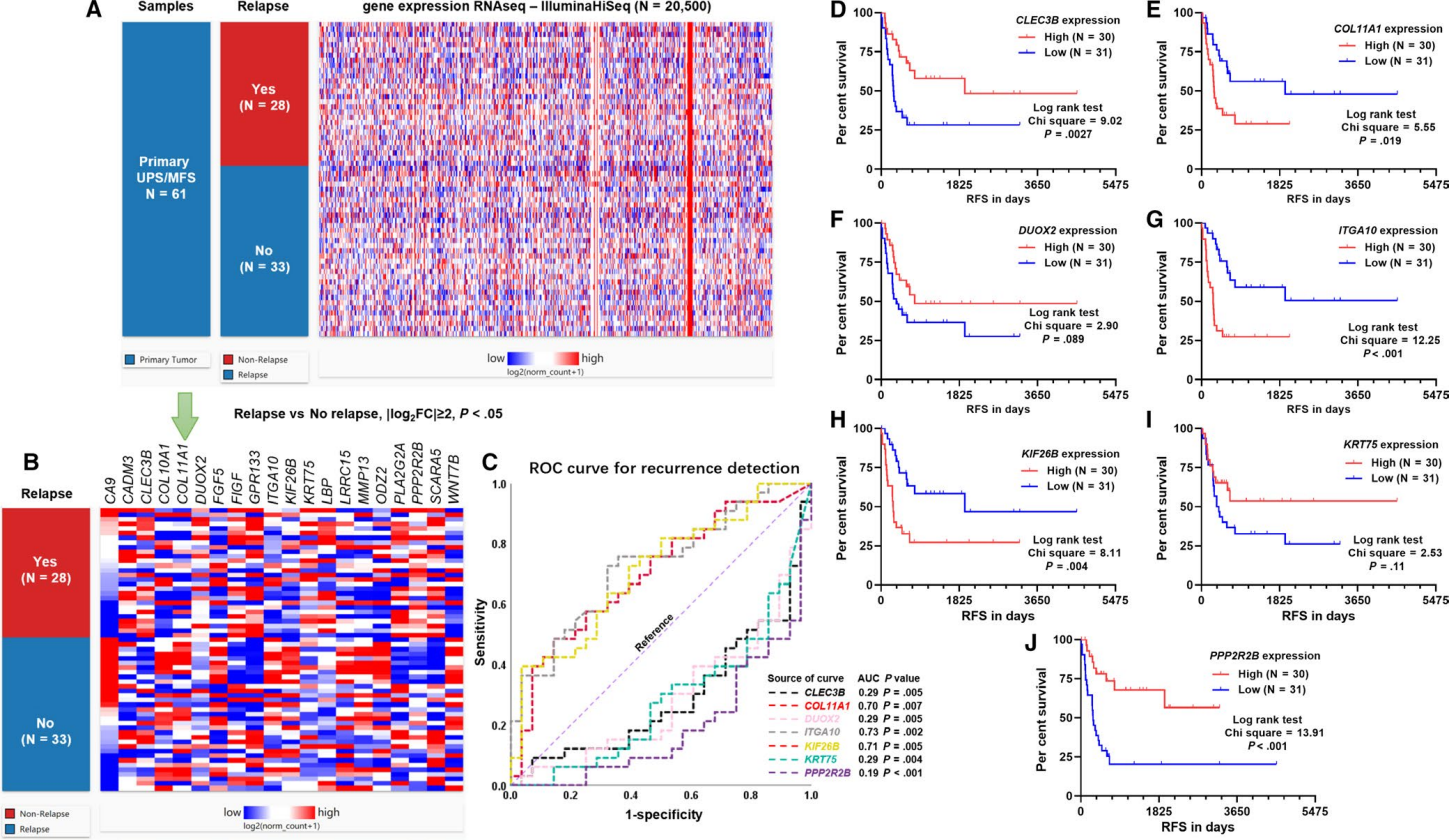
Systematic screening to identify relapse‐related genes in UPS/MFS. (A) Heatmap showing the expression profile of 20,500 genes measured by IlluminaHiSeq, between UPS/MFS cases with or without relapse. The dysregulated genes were screened by the criteria: Relapse *vs.* No relapse, |log_2_FC|≥2, *P* < .05. *p*‐value was calculated by performing unpair t test with Welch's correction. (B) Heatmap showing the expression profile of 20 dysregulated genes after screening. (C) ROC curves of the genes with an AUC ≥ 0.7 or ≤ 0.3, when predicting relapse. Relapse (1) was set as the status variable. (D‐J) Kaplan‐Meier curves of RFS of 61 UPS/MFS patients. The patients were separated into two groups according to the median expression of *CLEC3B, COL11A1, DUOX2, ITGA10, KIF26B, KRT75* and *PPP2R2B*

RNA‐seq data of 20 500 genes in TCGA‐SARC were downloaded from the UCSC Xena Browser. To obtain the list of dysregulated genes between cases with relapse (N = 28) and without relapse (N = 33), the following criteria were applied: absolute log2 fold change ≥ 2, Welch's t test *P*‐value < .05 (Figure 1A). After this screening process, 20 dysregulated genes were identified (Figure 1B). Then, we preliminarily analysed the prognostic value of these genes in terms of relapse by generating ROC curves. The AUC values of these genes were given in Table S1. Only the genes with AUC value ≥ 0.70 or ≤ 0.30 were considered as the candidate biomarkers (N = 7) (Figure 1C). The K‐M curves of RFS were generated by setting the median expression of the 7 genes individually. The log‐rank test showed that the group with high *CLEC3B* (3p21.31) or *PPP2R2B* (5q32) expression had significantly longer RFS (Figure 1D and J), while the group with high *COL11A1* (1p21.1)*, ITGA10* (1q21.1) or *KIF26B* (1q44) expression had significantly shorter RFS (Figure 1E, G and H). No significant difference was observed in the other two comparisons (Figure 1F,I).

### 3.2 Construction of prognostic gene signature for UPS/MFS

**Figure 2:**
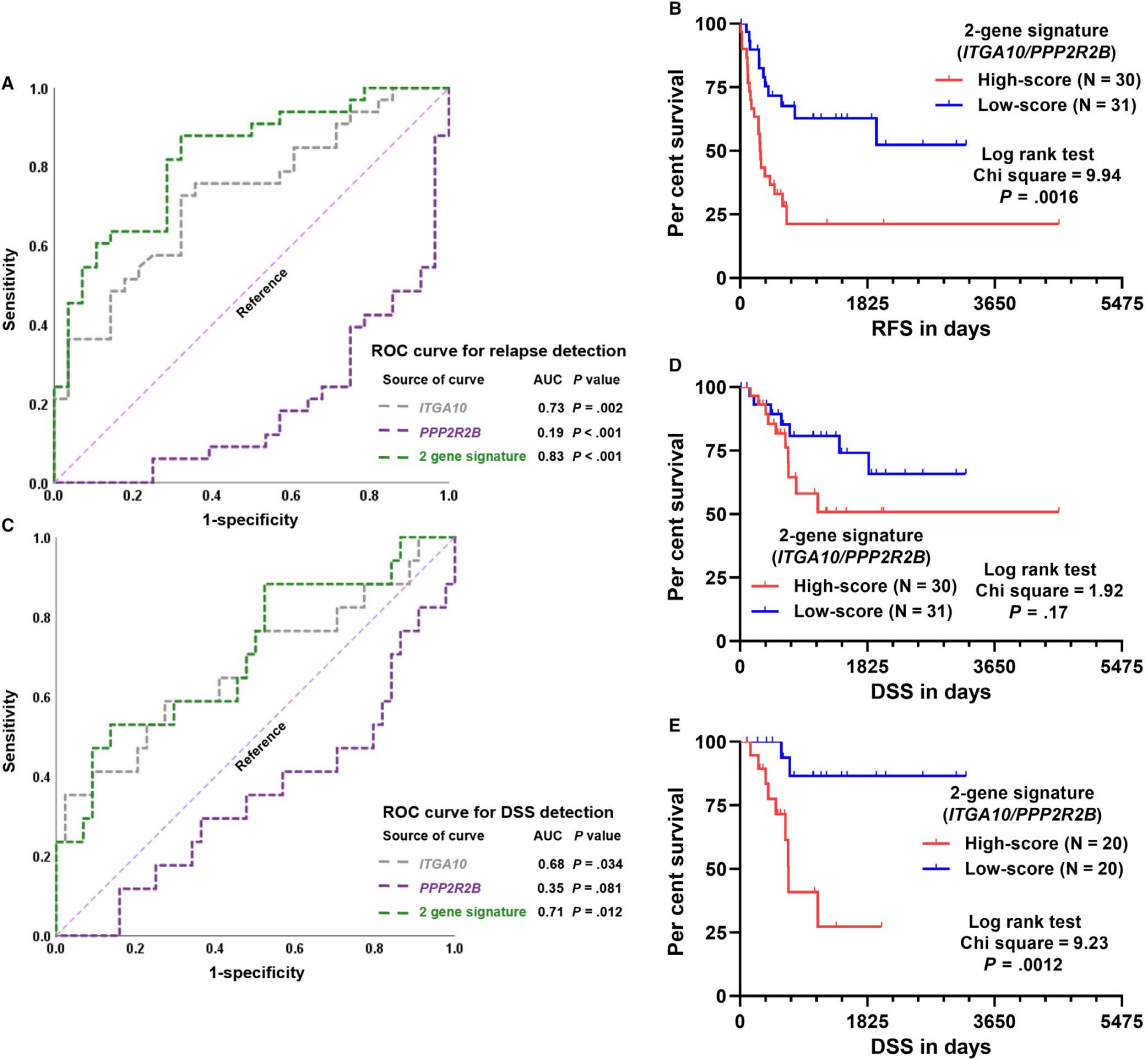
ROC curve and K‐M curve analysis of the prognostic value of the 2‐gene signature. (A and C) ROC curves of the predictive value of the 2‐gene signature for relapse (A) and DSS (C) detection. (B, D and E) Kaplan‐Meier curves of RFS (B) and DSS (D and E) of 61 UPS/MFS patients. The comparisons were performed between two groups separated by the median risk score (B and D) or between the first and third tertile of the risk score (E)

Then, the independent prognostic value of these genes was assessed. In univariate analysis, larger tumour size, with pharmaceutical drug adjuvant therapy, high FNCLCC grade, increased *COL11A1, ITGA10* and *KIF26B* expression*,* and decreased *CLEC3B, DUOX2, KRT75* and *PPP2R2B* were risk factors of shorter RFS (Table 1). Multivariate analysis showed that among the seven candidate genes, only *ITGA10* and *PPP2R2B* had independent prognostic value (*ITGA10*, HR: 1.399, 95%CI: 1.291‐1.629, *P* < .001; *PPP2R2B*, HR: 0.765, 95%CI: 0.632‐0.926, *P* = .006) after adjustment for other risk factors (Table 1). Therefore, only these two genes were included to construct a risk score model (2‐gene signature), with the following formula: risk score = 0.254*ITGA10+‐0.317*PPP2R2B. ROC curve analysis showed that the 2‐gene signature had an AUC value of 0.83 when predicting relapse, which was higher than that of *ITGA10* or *PPP2R2B* alone (Figure 2A). Patients were separated into two groups by setting the median value of the risk score as the cut‐off. K‐M analysis indicated a significant difference in RFS between high‐score and low‐score group (log‐rank test *P* = .0016, Figure 2B). Subgroup analysis confirmed effective relapse risk stratification of the 2‐gene signature in UPS and MFS patients, respectively (Figure S1A‐B). Multivariate analysis further confirmed the independent prognostic value of the 2‐gene signature as a continuous variable (HR: 2.966, 95%CI: 1.995‐4.410 *P* < .001), after adjustment for pathological tumour size, pharmaceutical drug adjuvant therapy and FNCLCC grade (Table 2). We then checked the potential prognostic value of the 2‐gene signature in terms of DSS. ROC curve showed that the 2‐gene signature had an AUC of 0.71 (Figure 2C). K‐M survival curve failed to identify a significant difference between the two groups by median separation (Figure 2D), but confirmed the difference between the first tertile and the third tertile (Figure 2E). By performing univariate and multivariate analysis, we found that the 2‐gene signature showed an independent prognostic value in DSS as a continuous variable (HR: 2.283, 95%CI: 1.358‐3.835, *P* = .002) (Table 3).

**Table 1:** Univariate and multivariate analysis of the prognostic value of relapse‐related genes in terms of RFS in UPS/MFS

Parameters	Univariate analysis				Multivariate analysis			
	*p*	HR	95% CI (lower/upper)		*p*	HR	95% CI (lower/upper)	
Age (Continuous)	0.514	0.991	0.963	1.019				
Gender								
Male (N = 30)		1.000						
Female (N = 31)	0.452	0.769	0.387	1.527				
Pathological tumour size (mm)	**<0.001**	1.094	1.040	1.151	**<0.001**	1.113	1.050	1.179
Residual tumour								
Yes (N = 20)		1.000						
No (N = 39)	0.122	0.571	0.281	1.161				
Radiation treatment adjuvant								
Yes (N = 34)		1.000						
No (N = 25)	0.899	1.047	0.516	2.123				
Pharmaceutical drug adjuvant								
Yes (N = 10)		1.000						
No (N = 38)	**0.023**	0.387	0.171	0.877				
FNCLCC grade								
1/2 (N = 18)		1.000						
3 (N = 43)	**0.038**	2.736	1.055	7.097				
*CLEC3B*	**0.001**	0.822	0.730	0.926				
*COL11A1*	**0.005**	1.119	1.035	1.210				
*DUOX2*	**0.010**	0.846	0.745	0.961				
*ITGA10*	**<0.001**	1.348	1.176	1.544	**<0.001**	1.399	1.201	1.629
*KIF26B*	**0.002**	1.205	1.072	1.354				
*KRT75*	**0.029**	0.872	0.771	0.986				
*PPP2R2B*	**<0.001**	0.692	0.578	0.828	**0.006**	0.765	0.632	0.926

Note

Multivariate analysis was conducted by forward: LR method.

Bold value indicates *P* < .05.

**Table 2:** Univariate and multivariate analysis of the prognostic value of the 2‐gene signature in terms of RFS in UPS/MFS

Parameters	Univariate analysis				Multivariate analysis			
	*p*	HR	95% CI (lower/upper)		*p*	HR	95% CI (lower/upper)	
Pathological tumour size (mm)	**<0.001**	1.094	1.040	1.151	**<0.001**	1.102	1.044	1.164
Pharmaceutical drug adjuvant therapy								
Yes (N = 10)		1.000						
No (N = 38)	**0.023**	0.387	0.171	0.877				
FNCLCC grade								
1/2 (N = 18)		1.000						
3 (N = 43)	**0.038**	2.736	1.055	7.097				
2‐gene signature (*ITGA10/PPP2R2B*)	**<0.001**	2.717	1.856	3.976	**<0.001**	2.966	1.995	4.410

Note

Multivariate analysis was conducted by forward: LR method.

Bold value indicates *P* < .05.

**Table 3:** Univariate and multivariate analysis of the prognostic value of the 2‐gene signature in terms of OS in UPS/MFS

Parameters	Univariate analysis				Multivariate analysis			
	*p*	HR	95% CI (lower/upper)		*p*	HR	95% CI (lower/upper)	
Age (Continuous)	0.919	1.002	0.963	1.043				
Gender								
Male (N = 30)		1.000						
Female (N = 31)	0.267	0.576	0.217	1.525				
Pathological tumour size (mm)	**<0.001**	1.155	1.076	1.241	**0.001**	1.142	1.058	1.233
Residual tumour								
Yes (N = 20)		1.000						
No (N = 39)	0.916	0.944	0.323	2.761				
Radiation treatment adjuvant								
Yes (N = 34)		1.000						
No (N = 25)	0.278	1.698	0.653	4.414				
Pharmaceutical drug adjuvant								
Yes (N = 10)		1.000						
No (N = 49)	0.648	0.768	0.247	2.388				
FNCLCC grade								
1/2 (N = 18)		1.000						
3 (N = 43)	0.362	1.786	0.513	6.219				
2‐gene signature (*ITGA10/PPP2R2B*)	**0.001**	1.895	1.315	2.731	**0.002**	2.283	1.358	3.835

Note

Multivariate analysis was conducted by forward: LR method.

Bold value indicates *P* < .05.

### 3.3 Analysis of potential mechanisms leading to dysregulated *ITGA10* and *PPP2R2B*

**Figure 3:**
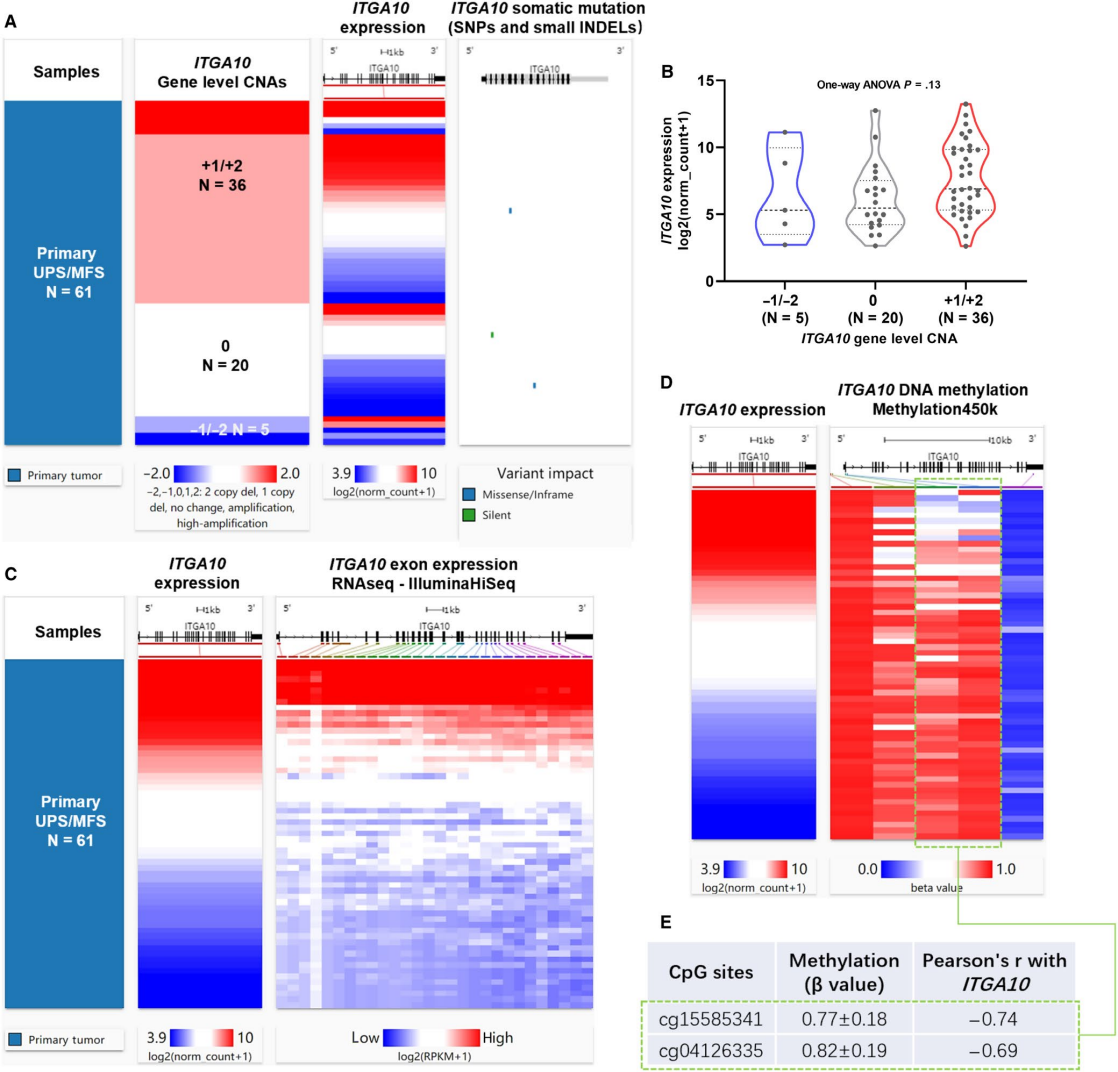
The association between gene‐level CNA/methylation and *ITGA10* expression. (A) Heatmap showing the correlation between *ITGA10* DNA CNAs, its expression and somatic mutations. (B) Plot chart showing the expression of *ITGA10* in different CNA groups. CNAs were defined as: copy neutral (0), low‐level copy gain (+1), high‐level amplification (+2), heterozygous loss (−1) or homozygous deletion (−2). (C) Heatmap showing the correlation between *ITGA10* expression and the expression of its exons. (D‐E) Heatmap (D) and statistical summary of the Pearson's correlation coefficient (at least moderately correlated, Pearson's r ≥ 0.4 or ≤‐0.4, E) between the methylation level (beta value) of CpG sites in *ITGA10* gene locus and *ITGA10* expression

Gene‐level CNA, somatic mutation and methylation data were extracted for dysregulation‐related analysis. We firstly checked *ITGA10* CNA status and corresponding gene expression. Among the 61 UPS/MFS cases, there were 36 amplification, 20 copy neutral and five deletion cases (Figure 3A). However, no significant difference in *ITGA10* expression was observed among the three groups (Figure 3B). There were 2 missense mutations (p.N336S and p.R668Q) and 1 silent mutation (p.G89G) (Figure 3A), suggesting that somatic mutation was not common in this gene. Then, we analysed the correlation between *ITGA10* expression and its exon transcription. Results showed that the transcription of all *ITGA10* exons showed strong positive correlations with *ITGA10* expression (Figure 3C). Transcript expression analysis showed that the two dominant transcripts of *ITGA10* (ENST00000369304.7 and ENST00000539363.2) in TCGA sarcoma cases contain exon 1 (Figure S2), suggesting that the promoter of *ITGA10* transcription locates before this exon. By checking the methylation of 5 CpG sites in *ITGA10* locus, we found two CpG sites (cg15585341 and cg04126335), the methylation of which showed strong negative correlations with *ITGA10* expression (Pearson's r <−0.6, Figure 3D‐E, green dotted frame). Cg15585341 locates within exon 1, while cg04126335 is in the first intron of *ITGA10* (Figure 3D), suggesting that their hypomethylation status might contribute to enhanced transcription of the gene.

**Figure 4:**
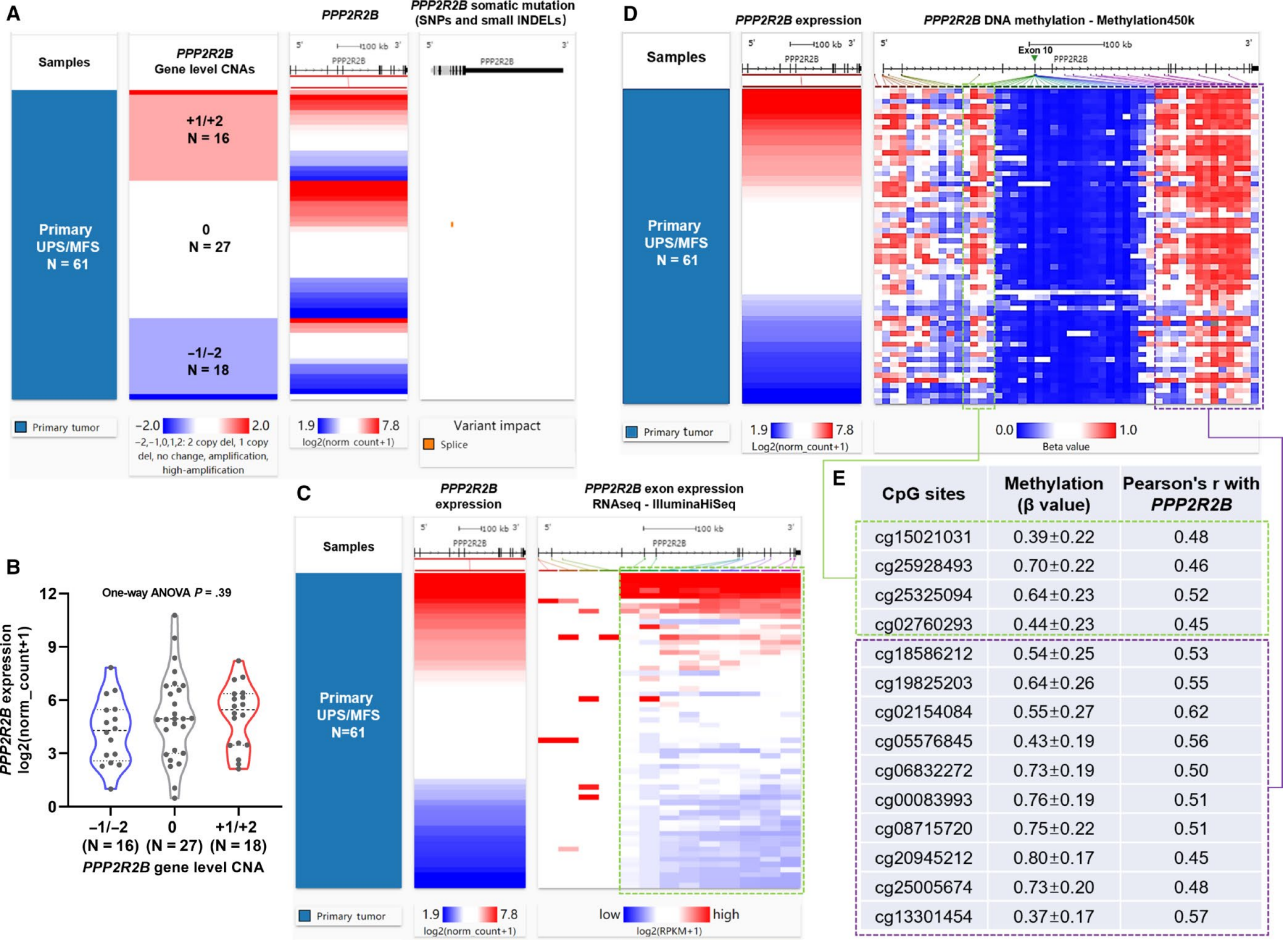
The association between gene‐level CNA/methylation and *PPP2R2B* expression. (A) Heatmap showing the correlation between *PPP2R2B* DNA CNAs, its expression and somatic mutations. (B) Plot chart showing the expression of *PPP2R2B* in different CNA groups. (C) Heatmap showing the correlation between *ITGA10* expression and the expression of its exons. (D‐E) Heatmap (D) and statistical summary of the Pearson's correlation coefficient (at least moderately correlated, Pearson's r ≥ 0.4 or ≤‐0.4, E) between the methylation level (beta value) of CpG sites in *PPP2R2B* gene locus and *PPP2R2B* expression

Similarly, *PPP2R2B* gene‐level CNA was irrelevant to its expression (Figure 4A‐B). One case with splice mutation (p.E56_splice) was detected (Figure 4A). By checking the correlation between *PPP2R2B* expression and its exon transcription, only a proportion of exons were positively correlated with its expression (Figure 4C, green dotted frame). *PPP2R2B* is a large gene that is around 500kb and contains at least 20 exons. Transcript expression analysis suggests that this gene encodes quite complex alternative transcripts in sarcoma tissues (Figure S3). Previous studies showed that the complex alternative transcription is at least partly driven by the promoter region flanking exon 7^18^, ^19^ (now exon 10 in UCSC Xena browser). This exon has multiple internal splicing sites, thereby generating several splice variants. Only the transcripts start from exon 10 to exon 21 were expressed in sarcoma. The two dominant transcripts (ENST00000394411.8 and ENST00000394409.7, Figure S3, red dotted frame) only contain 9 coding exons. *PPP2R2B* has well‐characterized expression in brain tissues.^19^, ^20^ Therefore, we compared its expression profile in sarcoma with normal brain tissues. In normal brain tissues, not only the transcripts start from exon 10, but also the transcripts start from exon 2/3 were expressed (Figure S3). These findings suggest that *PPP2R2B* has tissue‐specific transcriptional pattern.

By checking the methylation status of the CpG sites in *PPP2R2B* locus, we found the methylation of some CpG sites in two gene body regions (Figure 4D, green and purple dotted frames) showed at least moderate positive correlations with *PPP2R2B* expression (Figure 4E). cg15021031, cg25928493, cg25325094 and cg02760293 in the green dotted frame locate at the intron regions before exon 10, while the 10 CpG sites in purple dotted frame scatter across the introns after exon 10 (Figure 4D‐E).

### 3.4 The 2‐gene signature was associated with immune cell infiltration in UPS/MFS

**Figure 5:**
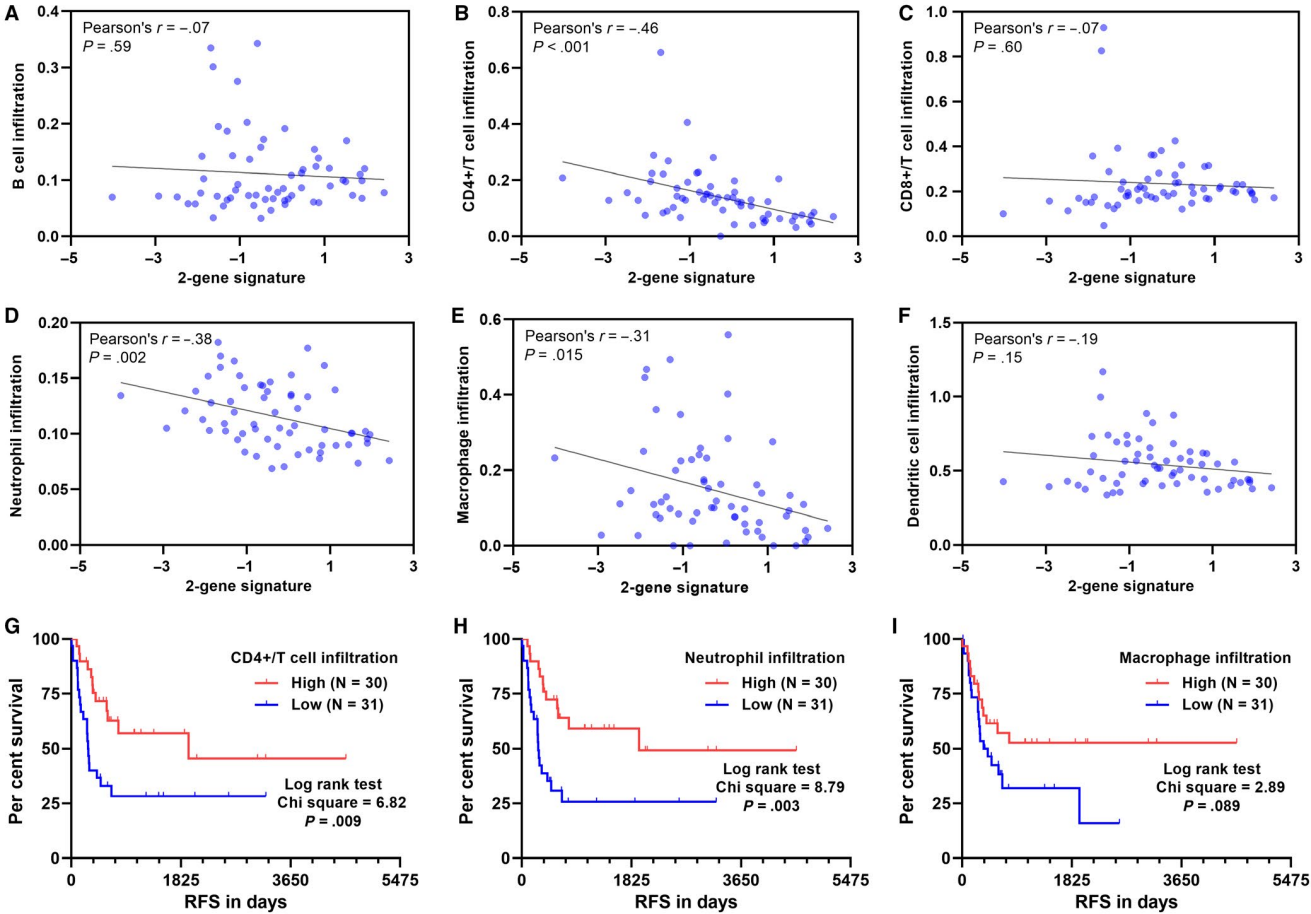
The association between the 2‐gene signature and immune cell infiltration in UPS/MFS. (A‐F) Pearson's correlation analysis between the 2‐gene signature and the infiltration of B cells (A), CD4 + T cells (B), CD8 + T cells (C), neutrophils (D), macrophages (E) and dendritic cells (F) in UPS/MFS cases. G‐I. Kaplan‐Meier curves of RFS 61 UPS/MFS patients separated by median infiltration of CD4 + T cells (G), neutrophils (H) and macrophages (I)

The correlations between the 2‐gene signature and the infiltration of six immune cell types in UPS/MFS were estimated. Regression analysis found a moderate negative correlation between the 2‐gene signature and CD4 + T cell infiltration (Pearson's *r* = −0.46, Figure 5B) and weak negative correlations between the 2‐gene signature and neutrophil/macrophage infiltration (Figure 5D‐E). No significant correlation was observed in B cells, CD8 + cells and dendritic cells (Figure 5A, 5 and 5). K‐M survival curves showed that high level of CD4 + T cell infiltration and neutrophil infiltration were associated with significantly better RFS (Figure 5G‐H). But the difference between the high and low macrophage infiltration groups was not significant (Figure 5I).

Then, we assessed the potential prognostic significance of immune cell infiltrations. Univariate analysis suggested that CD4+/T cell, neutrophil and macrophage infiltration might be indicators of favourable RFS, but no association was observed in terms of DSS (Table S2). Then, we conducted a ROC analysis to compare the prognostic value of immune cell infiltrations and the 2‐gene signature. Results showed that the 2‐gene signature had the highest AUC value, suggesting a better prognostic value (Figure S4).

### 3.5 The potential involvement of *ITGA10* AND *PPP2R2B* in AKT/MTOR signalling

**Figure 6:**
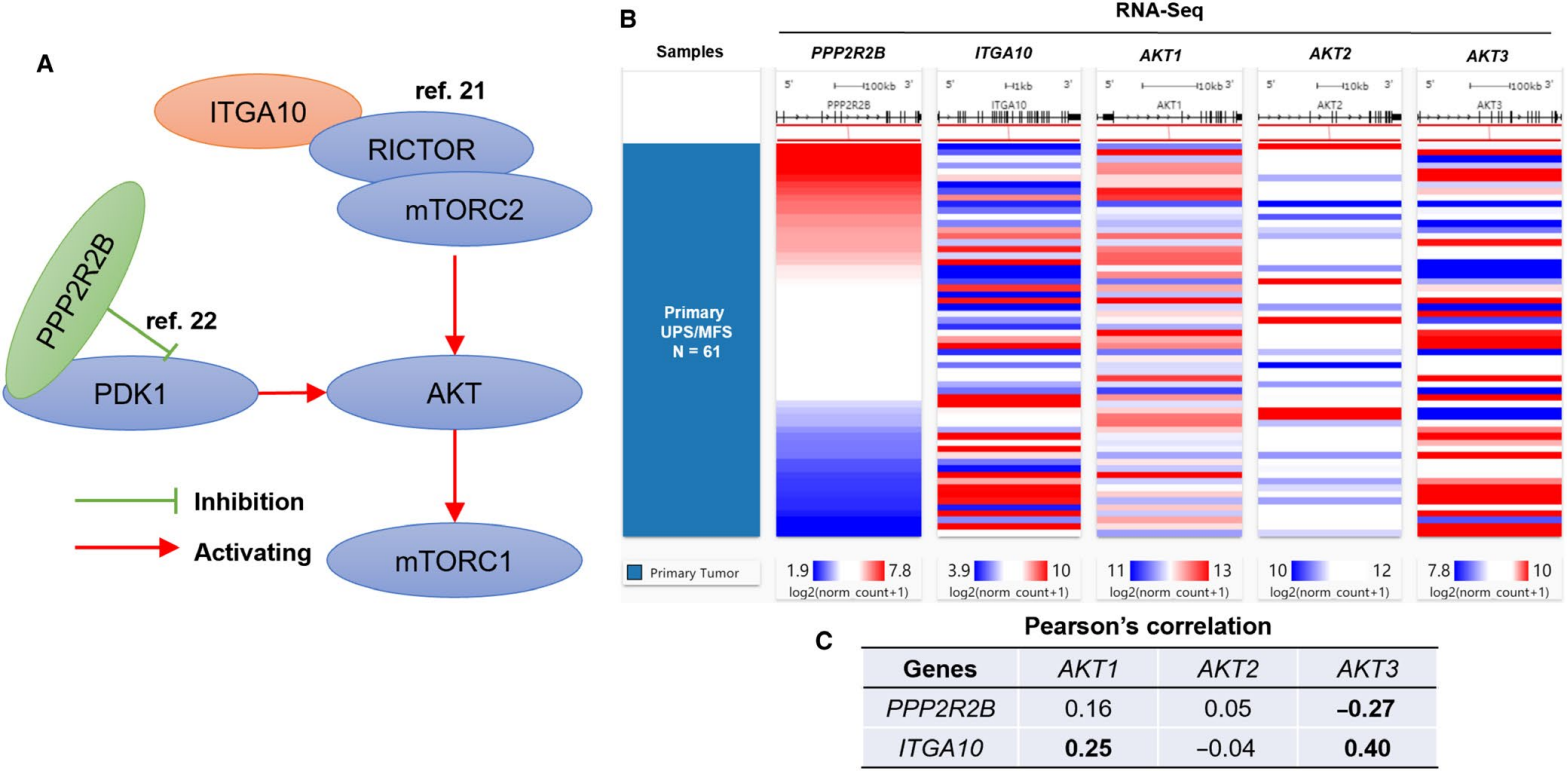
The potential involvement of *ITGA10* and *PPP2R2B* in AKT/mTOR signalling pathway. (A) A schematic image showing the potential involvement of *PPP2R2B* and *ITGA10* in AKT/mTOR signalling. (B‐C) Heatmap (B) and statistical chart (C) showing the correlation between the expression of *PPP2R2B/ITGA10* and AKT1/2/3

Available evidence showed that *ITGA10* and *PPP2R2B* might be important modulators in AKT/mTOR signalling. *ITGA10* encodes integrin‐α10 in human genome. Integrin‐α10 has tumour‐specific physical association with TRIO and RICTOR, the expression of which promotes sarcoma cell survival via activating the RAC/PAK and AKT/rapamycin (mTOR) complex 1 (mTORC1) signalling^21^ (Figure 6A). *PPP2R2B* encodes the regulatory B55b subunit of protein phosphatase 2A (PP2A). It directly interacts with PDK1 and suppresses PDK1 activation via inhibiting its phosphorylation and recruitment from the cytosol to the plasma membrane^22^ (Figure 6A). Therefore, both *ITGA10* and *PPP2R2B* might act as upstream regulators of AKT. To validate the potential influence of *ITGA10* and *PPP2R2B* on AKT expression in UPS/MFS tissues, we calculated their correlation coefficients with *AKT1, AKT2* and *AKT3* expression. Results showed *ITGA10* was positively correlated with *AKT1* and *AKT3* expression (Pearson's *r* > .2), while *PPP2R2B* was negatively correlated with *AKT3* expression (Pearson's *r* < −.2) (Figure 6B‐C).

## 4 DISCUSSION

Integrin‐α10 is an integral transmembrane glycoprotein that participates in cell adhesion as well as cell surface‐mediated signalling. Its dysregulation and oncogenic effects were observed in lung cancer,^23^ prostate cancer^24^ and MFS.^21^ PP2A is a serine/threonine (Ser/Thr) protein phosphatase, which has a heterotrimeric structure formed by a catalytic subunit (PP2Ac), an A subunit and a member of the B subunit family.^25^ The tumour suppressor role of PP2A has been reported in several types of human cancer, such as colorectal cancer,^26^ non‐small cell lung cancer,^27^ leukemia^28^ and sarcoma.^29^ Using data in other TCGA cohorts, we found that *ITGA10* might be associated with unfavourable RFS in LAML, PRAD and UVM and unfavourable OS in KIRP, LGG and THCA. Besides, its expression was related to favourable RFS in THCA and favourable OS in SKCM (Figure S5). In comparison, *PPP2R2B* might be associated with favourable RFS in ACC, BLCA, CHOL, PAAD and SKCM, as well as favourable OS in ACC, BLCA, BRCA, LGG, LUAD, OV, PAAD and SKCM. However, it might be an indicator of unfavourable OS in KRIP, THCA and UVM (Figure S5). These findings suggest that the prognostic value of these two genes might be cancer‐specific. The systematic profiling in this study found that *ITGA10* and *PPP2R2B* expression could form a 2‐gene signature that has good prognostic value (AUC = 0.83) in terms of RFS in UPS/MFS. The 2‐gene signature had an independent prognostic value in both RFS and DSS, as a continuous variable.

By checking genomic alterations of these two genes, we found that CNA was irrelevant to their expression. In comparison, DNA methylation showed a profound effect on their expression. Two CpG sites near the promoter region of *ITGA10* showed strong negative correlations with *ITGA10* expression. Some previous studies suggest that DNA hypermethylation was associated with decreased *PPP2R2B* expression in breast cancer,^30^ nasopharyngeal carcinoma cells^31^ and glioma.^32^ However, in this study, we observed that the methylation of some CpG sites in two gene body regions of *PPP2R2B* showed at least moderate positive correlations with *PPP2R2B* expression. There are emerging studies showed that gene body methylation might be positively correlated with gene expression, which is a different regulatory effect compared with promoter methylation.^33^ The exact mechanisms of the positive correlation have not been fully revealed. Some potential mechanisms, including inhibiting the initiation of intragenic promoters and modulating the transcription activity of repetitive DNAs within the transcriptional unit and/or by influencing transcriptional elongation.^34^, ^35^ Gene body demethylation might also result in nucleosome destabilization in the transcribed region and thus increasing transcription efficiency.^33^, ^36^, ^37^ In addition, it might contribute to the formation of borders at enhancers or promoters, thereby enhancing the transcription of specific transcripts.^38^, ^39^, ^40^

Among STS subtypes, UPS/MFS has the highest median macrophage infiltration and the infiltration of immature dendritic cells was positively correlated with DSS.^3^ These findings suggest that UPS/MFS may have immunologic mutated protein targets and thus respond to immune checkpoint therapy.^3^, ^41^ In the SARC028 trial of pembrolizumab (a PD‐1 inhibitor), four out of 10 UPS patients had responses to the drug.^42^ These findings suggest that patients with UPS/MFS might benefit from immunotherapy. In this study, we found that the 2‐gene signature had a moderate negative correlation with CD4 + T cell infiltration. We also confirmed that a high level of CD4 + T cell infiltration was associated with significantly better RFS. Therefore, we infer that the 2‐gene signature might also predict the infiltration of CD4 + T cells. However, the predictive value of this signature in immunotherapy responses should be studied in the future.

A series of previous studies reported that activation of the AKT/mTOR signalling is associated with pathology of UPS/MFS, such as higher histologic grade and progression.^21^, ^43^, ^44^ Small molecular inhibitors of RAC and mTORC significantly suppress the proliferation of MFS cells in vitro.^21^ Coinhibition of PI3K, mTOR and IGF1R shows synergic effects on inhibiting UPS cell proliferation, invasion and migration both in vitro and in a xenograft model.^44^ ITGA10 binds with TRIO and RICTOR and subsequently activates the RAC/PAK and AKT/mTORC1 signalling.^21^ Inhibiting *ITGA10* expression suppresses the proliferation and reduce survival of MFS cells, but has no influence on normal mesenchymal cells.^21^ PPP2R2B interacts with PDK1 and suppresses its activation.^22^ Based on the 61 UPS/MFS cases, we found positive correlations between *ITGA10* and *AKT1*/*AKT3* expression, as well as a negative correlation between *PPP2R2B* and *AKT3* expression. These findings suggest that both *ITGA10* and *PPP2R2B* are involved in the mTOR signalling pathway, but may have opposite regulatory effects. These mechanisms also help to explain the association between the 2‐gene signature and unfavourable survival of UPS/MFS.

This study also has some limitations. Firstly, no validation cohort was used to verify the prognostic value of the 2‐gene signature. Besides, the exact mechanisms involved in *ITGA10/PPP2R2B* dysregulation were not explored by molecular studies. Future studies are required to figure out these issues.

## 5 CONCLUSIONS

The systematic screening in this study found a 2‐gene signature based on *ITGA10* and *PPP2R2B* expression had a potential good prognostic value in RFS and DSS in patients with UPS/MFS. DNA methylation might be a critical mechanism leading to their dysregulation, but it might have opposite regulations on the two genes. Furthermore, this signature has a potential predictive value in estimating CD4 + T cell infiltration. Both *ITGA10* and *PPP2R2B* are involved in the mTOR signalling pathway, but showing opposite regulatory effects. Considering the potential benefits of immunotherapy for UPS/MFS patients, it is imperative to explore the predictive value of this signature in immunotherapeutic responses in the future.

## CONFLICT OF INTEREST

The authors confirm that there are no conflicts of interest.

## AUTHOR CONTRIBUTIONS

Conceptualization, QSH and SJZ; Methodology, QSH, SJZ and XFH; Software, SJZ and XFH; Validation, HZ and SSH; Formal Analysis, QSH and SJZ; Data Curation, QSH, SSH and YBW; Writing‐Original Draft Preparation, QSH and SJZ; Writing‐Review & Editing, QSH and YBW; Supervision, SSH and YBW.

## Data Availability

All data used in this study were included in the manuscript and supplementary materials.
