# Supplementary figures and images for: Systematic screening identifies a 2‐gene signature as a high‐potential prognostic marker of undifferentiated pleomorphic sarcoma/myxofibrosarcoma
Source: J Cell Mol Med. 2019 Nov 19;24(1):1010–21. doi: 10.1111/jcmm.14814 (PMC6933343; doi:10.1111/jcmm.14814)

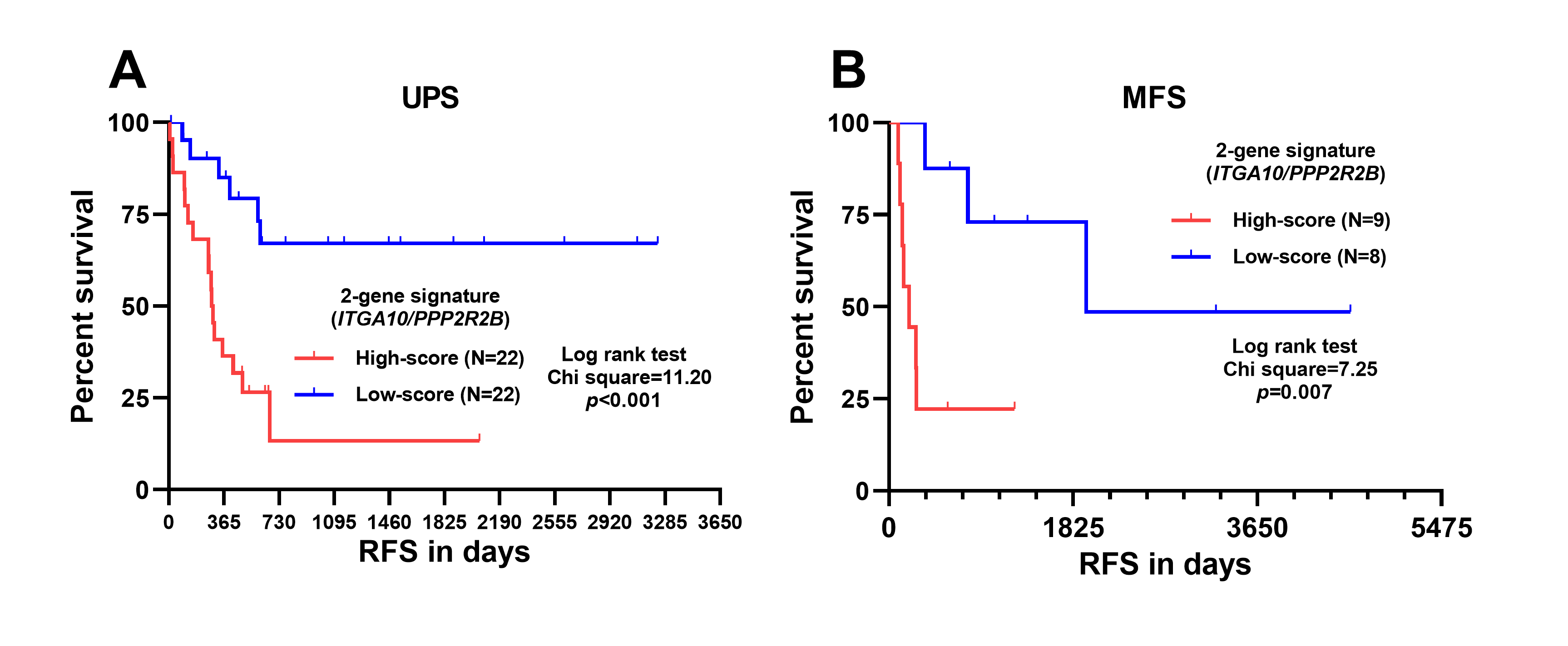

Supplement: Supplementary file 1 [file JCMM-24-1010-s001.tif]

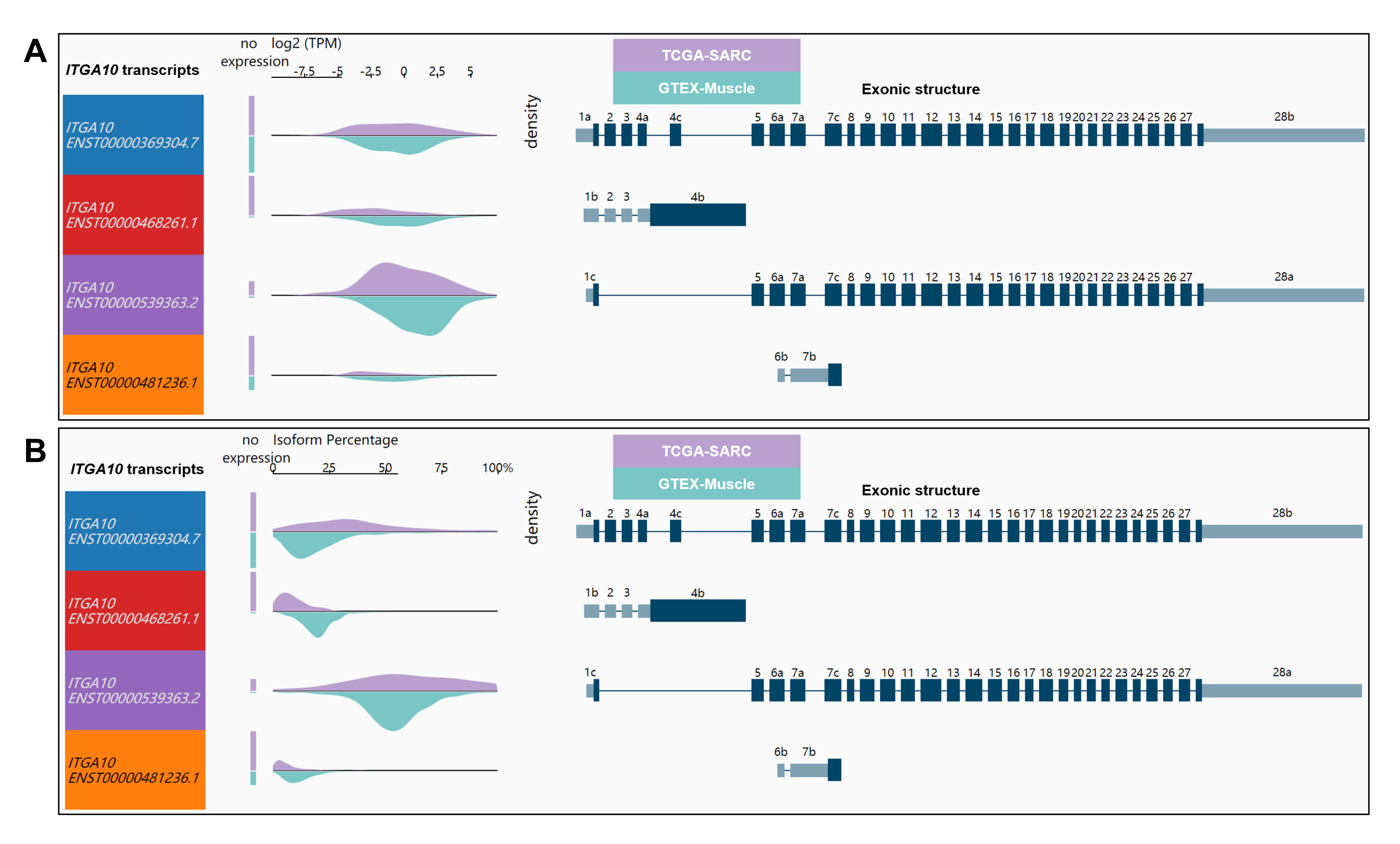

Supplement: Supplementary file 2 [file JCMM-24-1010-s002.tif]

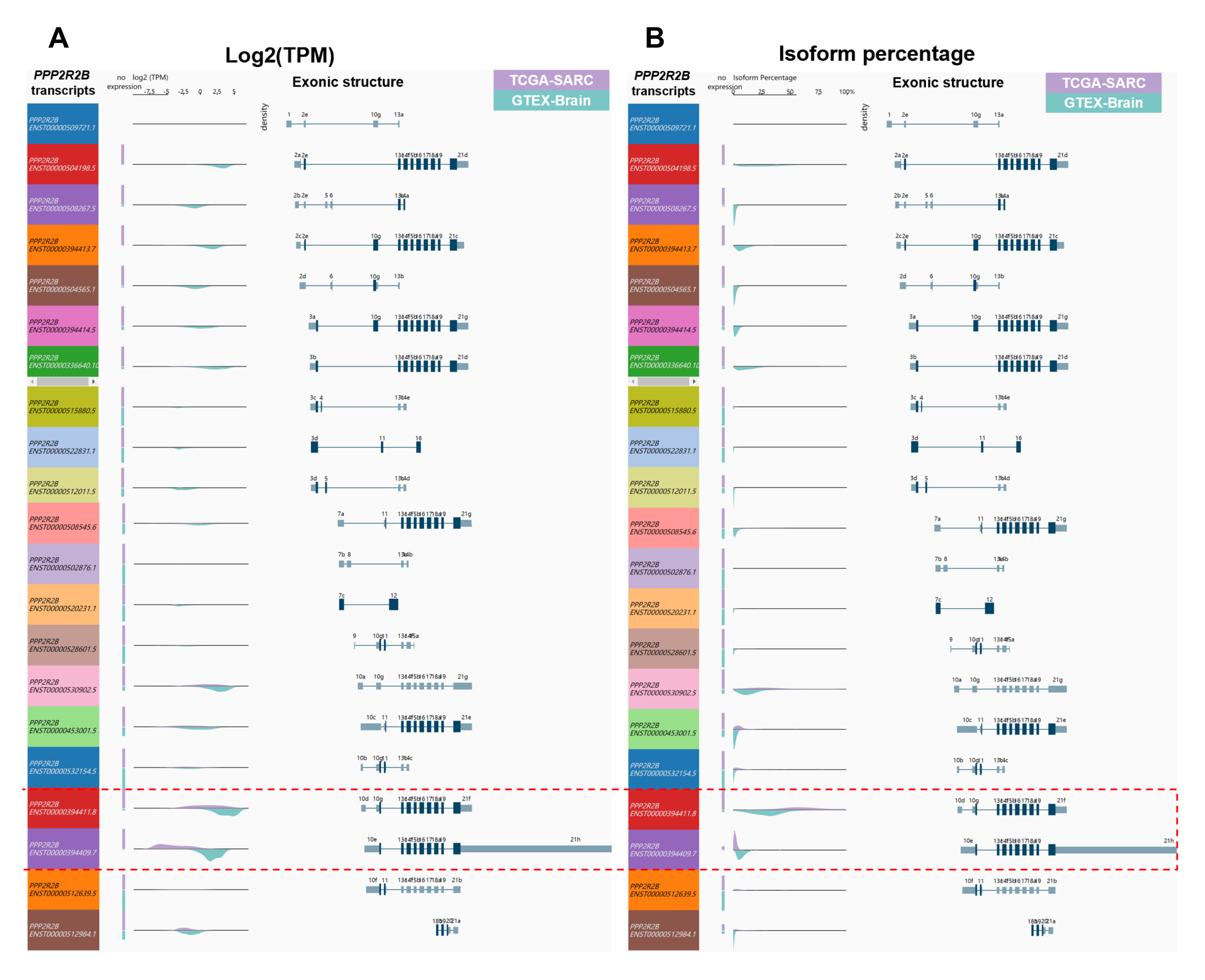

Supplement: Supplementary file 3 [file JCMM-24-1010-s003.tif]

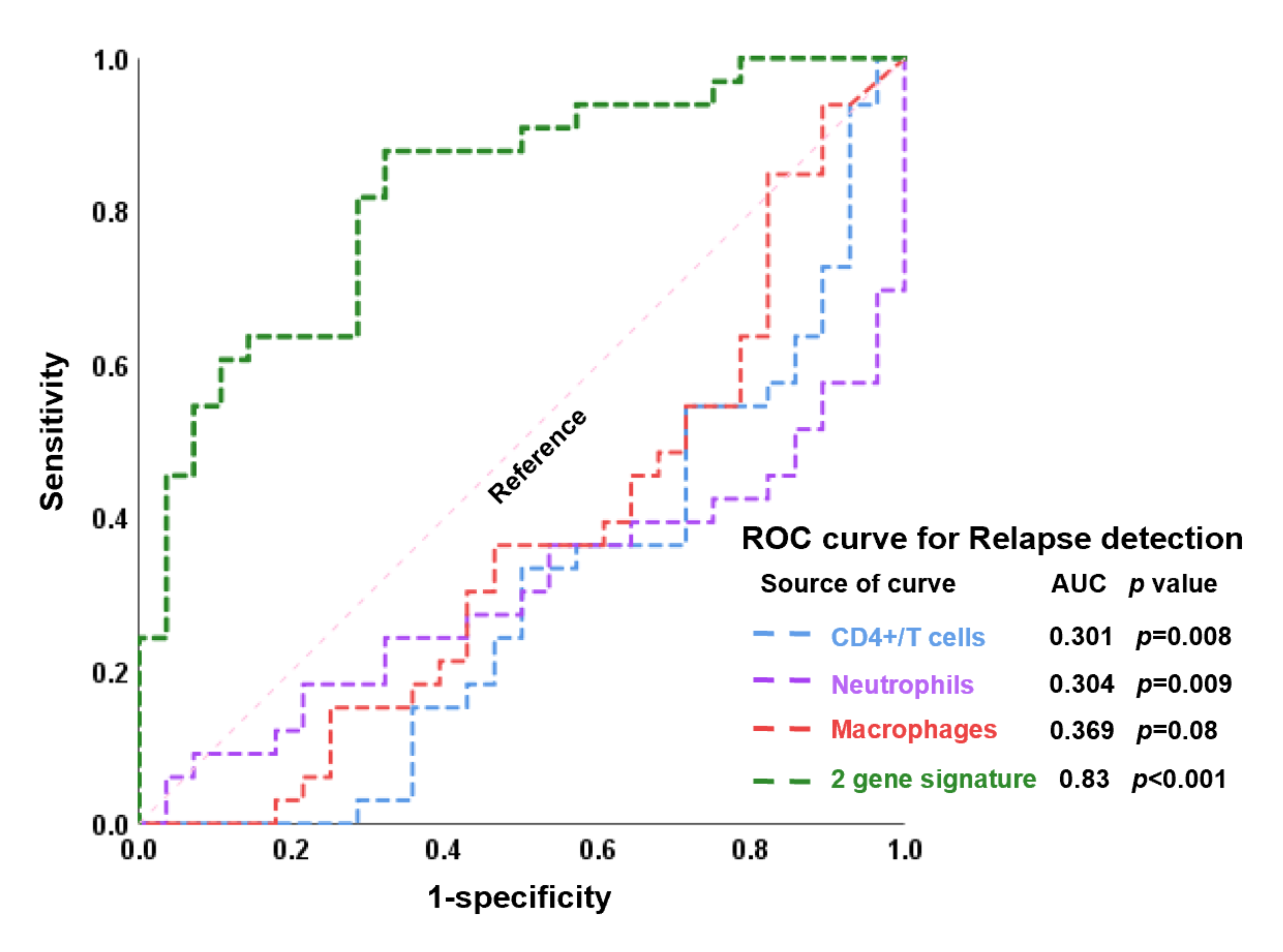

Supplement: Supplementary file 4 [file JCMM-24-1010-s004.tif]

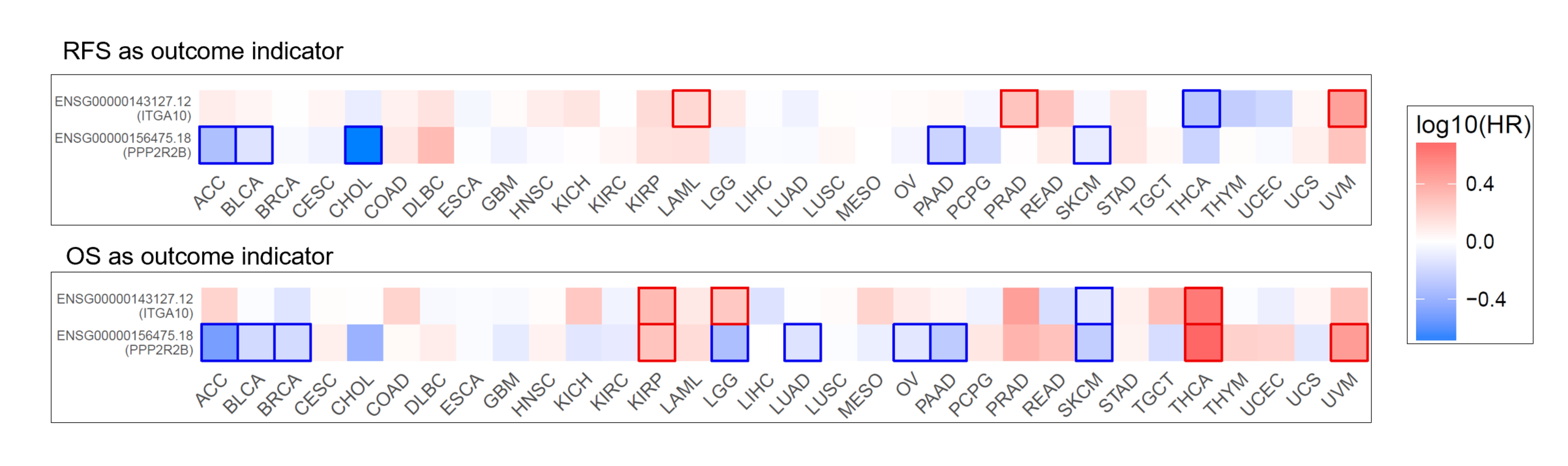

Supplement: Supplementary file 5 [file JCMM-24-1010-s005.tif]
