# Supplementary material for: Systematic screening identifies a 2‐gene signature as a high‐potential prognostic marker of undifferentiated pleomorphic sarcoma/myxofibrosarcoma
Source: J Cell Mol Med. 2019 Nov 19;24(1):1010–21. doi: 10.1111/jcmm.14814 (PMC6933343; doi:10.1111/jcmm.14814)
Supplement: Supplementary file 7 [file JCMM-24-1010-s007.docx]

**Supplementary Table 2. Univariate analysis of the prognostic value of the abundance of tumor-infiltrating immune cells in UPS/MFS**

| **Parameters** | **Univariate analysis** | | | |
| --- | --- | --- | --- | --- |
|  | ***p*** | **HR** | **95%CI (lower/upper)** | |
| **RFS** |  |  |  |  |
| **B cell** | 0.814 | 0.549 | 0.004 | 82.093 |
| **CD4+/T cell** | **0.002** | <0.001 | <0.001 | 0.040 |
| **CD8+/T cell** | 0.376 | 0.273 | 0.016 | 4.823 |
| **Neutrophil** | **0.005** | <0.001 | <0.001 | 0.001 |
| **Macrophage** | **0.027** | 0.024 | 0.001 | 0.659 |
| **Dendritic cell** | 0.262 | 0.275 | 0.029 | 2.624 |
| **DSS** |  |  |  |  |
| **B cell** | 0.972 | 0.873 | <0.001 | 1524.010 |
| **CD4+/T cell** | 0.097 | 0.001 | <0.001 | 3.409 |
| **CD8+/T cell** | 0.189 | 0.010 | <0.001 | 9.655 |
| **Neutrophil** | 0.100 | <0.001 | <0.001 | 27.896 |
| **Macrophage** | 0.251 | 0.081 | 0.001 | 5.930 |
| **Dendritic cell** | 0.417 | 0.241 | 0.008 | 7.480 |
